# The Evolution of *HoxD-11* Expression in the Bird Wing: Insights from *Alligator mississippiensis*

**DOI:** 10.1371/journal.pone.0003325

**Published:** 2008-10-03

**Authors:** Alexander O. Vargas, Tiana Kohlsdorf, John F. Fallon, John VandenBrooks, Günter P. Wagner

**Affiliations:** 1 Department of Ecology and Evolutionary Biology, Yale University, New Haven, Connecticut, United States of America; 2 Department of Anatomy, University of Wisconsin-Madison, Madison, Wisconsin, United States of America; 3 Department of Geology and Geophysics, Yale Peabody Museum of Natural History, New Haven, Connecticut, United States of America; Paleontological Institute, Russian Federation

## Abstract

**Background:**

Comparative morphology identifies the digits of the wing of birds as 1,2 and 3, but they develop at embryological positions that become digits 2, 3 and 4 in other amniotes. A hypothesis to explain this is that a homeotic frame shift of digital identity occurred in the evolution of the bird wing, such that digits 1,2 and 3 are developing from embryological positions 2, 3 and 4. Digit 1 of the mouse is the only digit that shows no late expression of *HoxD-11*. This is also true for the anterior digit of the bird wing, suggesting this digit is actually a digit 1. If this is the case, we can expect closer relatives of birds to show no *HoxD-11* expression only in digit 1. To test this prediction we investigate *HoxD-11* expression in crocodilians, the closest living relatives of birds.

**Methodology/Principal Findings:**

Using degenerate primers we cloned a 606 nucleotide fragment of exon 1 of the alligator *HoxD-11* gene and used it for whole-mount in-situ detection in alligator embryos. We found that in the pentadactyl forelimbs of alligator, as in the mouse, late expression of *HoxD-11* is absent only in digit 1.

**Conclusions/Significance:**

The ancestral condition for amniotes is that late-phase *HoxD-11* expression is absent only in digit 1. The biphalangeal morphology and lack of *HoxD-11* expression of the anterior digit of the wing is like digit 1 of alligator and mouse, but its embryological position as digit 2 is derived. *HoxD-11* expression in alligator is consistent with the hypothesis that both digit morphology as well as *HoxD-11* expression are shifted towards posterior in the bird wing.

## Introduction

**Figure 1:**
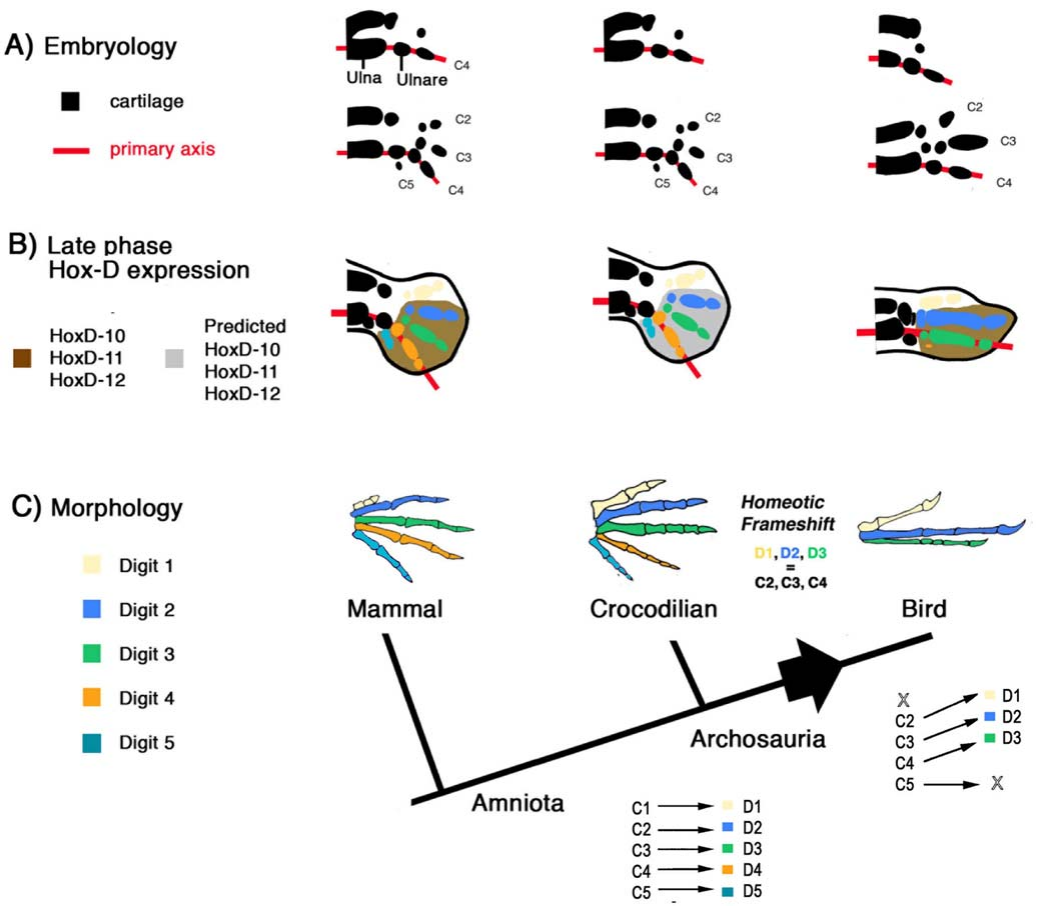
Three levels to the avian digit homology problem: embryology, gene expression, and morphology. A) Embryology: In pentadactyl amniotes like mammals and crocodilians, the primary axis of cartilage formation (red line) always develops into digit 4. Embryological condensations in this figure are labeled C1–C5 based on their spatial relation to the primary axis (C4). B) Gene expression (Late phase): In the mouse, expression of *HoxD10-D12* is absent only in digit 1. In the chicken, expression is absent in the anterior digit, but it is one position closer to the primary axis, at the embryological position of C2. C) Comparative morphology: The wing digits are morphologically 1, 2, and 3. The hypothesis of a homeotic frame shift proposes that digits 1,2 and 3 have all shifted one embryological position towards posterior in the evolution of the bird line, such that digits D1, D2 and D3 (color coded: cream, blue and green) in the wing develop from the embryological positions C2, C3 and C4. If the frame shift hypothesis is correct, we expect to find that HoxD gene expression in crocodilians will be absent only at embryological position C1.

**Figure 2:**
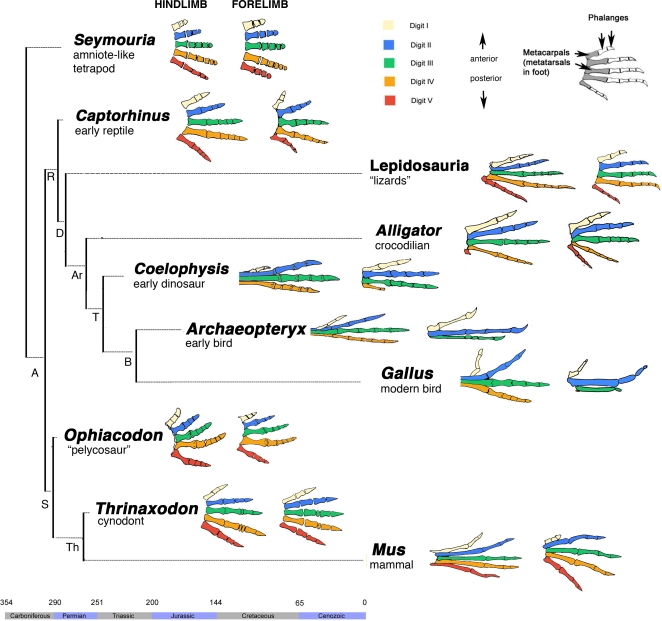
The evolution of digit morphology. The forelimb and hind limbs of representative taxa illustrate the history of digit morphology in the lineages leading to the taxa compared in this study, the chicken (*Gallus gallus*), alligator (*Alligator mississippiensis*) and mouse (*Mus musculus*). The digits of early birds like *Archaeopteryx*, are specifically similar to digits 1, 2, and 3 of crocodilians, presenting 2,3 and 4 phalanges on each digit, respectively (node Ar). We arrive at the same conclusion if we compare *Archaeopteryx* to early dinosaurs, lizards, and early branches of amniotes (such as *Captorhinus*, *Ophiacodon*). No comparative morphological evidence has been presented for a 2,3,4 identification of wing digits. Molecular phylogenies confirm the relationships shown in this figure [3], [33]. Maximally parsimonious inference of morphological history is done following the method in [34]. The nodes of the tree are labeled for corresponding clades: A) Amniota R) Reptilia, D) Diapsida, Ar) Archosauria, T) Theropoda, B) Birds S) Synapsida Th) Therapsida. A geological time scale indicates the approximate time of lineage divergence.

The identity of the digits of the bird wing is a classic problem of evolutionary biology, born out of apparently contradictory developmental and morphological evidence. If we follow the criterion of homology by embryological position of origin, we find that the wing digits develop from embryological positions corresponding to those of digits 2, 3 and 4 of crocodilians [1], [2]. Crocodilians are bird's closest living relatives [3] and thus the optimal reference point for developmental comparisons to the bird wing. In the alligator forelimb (as in mouse) the first cartilaginous digital condensation to form is spatially in line with the ulnare and ulna (Figure 1A, top row), and develops into digit 4 (Figure 1 A, bottom row). The spatial alignment of these elements is referred to as the “primary axis”, indicated by a red line in Figure 1. In the wing, the primary axis develops into the posterior digit, indicating the digits develop at positions 2, 3 and 4 [1], [2] (Figure 1A). However, the wing digits of early birds like *Archaeopteryx* are morphologically similar to digits 1, 2, and 3 of crocodilians, presenting 2, 3 and 4 phalanges, respectively (Figure 2). We arrive at the same conclusion if we compare *Archaeopteryx* to early dinosaurs, lizards, and even early branches of amniotes (Figure 2, See *Captorhinus*, *Ophiacodon*). Wing digits are labeled 1,2,3 in the fields of phylogenetic systematics and comparative anatomy [4], [5], [6], [7] As an explanation to this apparent contradiction with the embryological evidence, Wagner and Gauthier [8] suggested that a homeotic frame shift of digital identity had occurred in the evolution of the bird wing, such that in birds digits 1, 2 and 3 develop from embryological positions 2, 3 and 4 (Figure 1C).

Consistent with this hypothesis, the embryological position of HoxD gene expression appears to be shifted in the bird wing. The posterior HoxD genes (i.e. *HoxD-10*, *HoxD-11*, *HoxD-12*, and *HoxD-13*) are well known for their expression and function in developing digits [9], [10]. In the bird wing *HoxD-10*, *-11 and -12* are absent only at the most anterior digit [11], [12] (embryological position 2, Figure 1B). Because the same is true only for digit 1 of the mouse [13], Vargas and Fallon [14] argued that HoxD gene expression in the wing suggests a digit 1 develops at the embryological position of digit 2. If the comparison of digit 1 of the mouse to the anterior wing digit is correct, we should expect closer relatives of birds to show no expression of these genes only in digit 1 (The predicted expression for alligator is shown in gray shading in Figure 1B). If we do not assume a frame shift, but rather that wing digits develop directly into digits 2,3 and 4, expression in crocodilian forelimbs could be absent in digit 2. To test these predictions, we investigate *HoxD-11* expression in crocodilians (bird's closest living relatives). If expression in crocodilians is not uniquely absent in digit 1 (as in mouse), *HoxD-11* would provide no support for the homeotic frame shift hypothesis. We cloned a fragment of exon 1 of *HoxD-11* of the crocodilian *Alligator mississippiensis* and observed its transcription in developing digits. We found that, as in the mouse, in alligator forelimbs *HoxD-11* mRNA is absent only at digit 1. We discuss the relevance of this result for the hypothesis of a homeotic frame shift in the bird wing.

## Results

### Cloning and sequence analysis

**Figure 3:**
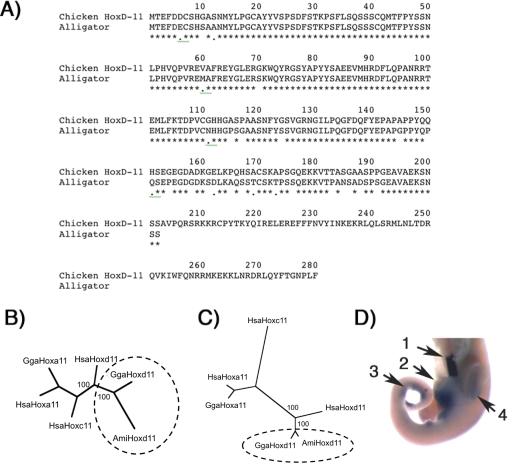
Identification of the alligator *HoxD-11* exon 1 sequence. (EU597806): A) alignment of the deduced alligator amino acid sequence with the *HoxD-11* sequence of chicken. In exon 1 the amino acid sequence conservation is 87%. B) Maximum parsimony tree of the aligned exon 1 amino acid sequences from *HoxD-11*, *HoxA-11* and *HoxC-11* sequences. C) Neighbor joining tree of the aligned exon 1 amino acid sequences from *HoxD-11*, *HoxA-11* and *HoxC-11* sequences. B+C) The numbers at the internal branches represent bootstrap support values. Note that the alligator *HoxD-11* sequence, AmiHoxd11, forms a well-supported clade with the human, HsaHoxd11, and the chicken, GgaHoxd11, *HoxD-11* sequences, confirming that the alligator sequence is a *HoxD-11* ortholog. Furthermore, the alligator sequence is more closely related to the chicken sequence than to the mouse sequence, as expected based on the accepted species phylogeny. D) Expression of the alligator sequence in a stage 12 alligator embryo. The embryo is dissected to show the sharp anterior limit of hindgut expression (1). Expression is also present in genital tubercule (2), distal tail (3) and limb buds (4), all known expression domains of *HoxD-11* in chicken and mouse that confirm the alligator sequence is a homolog of *HoxD-11*.

**Figure 4:**
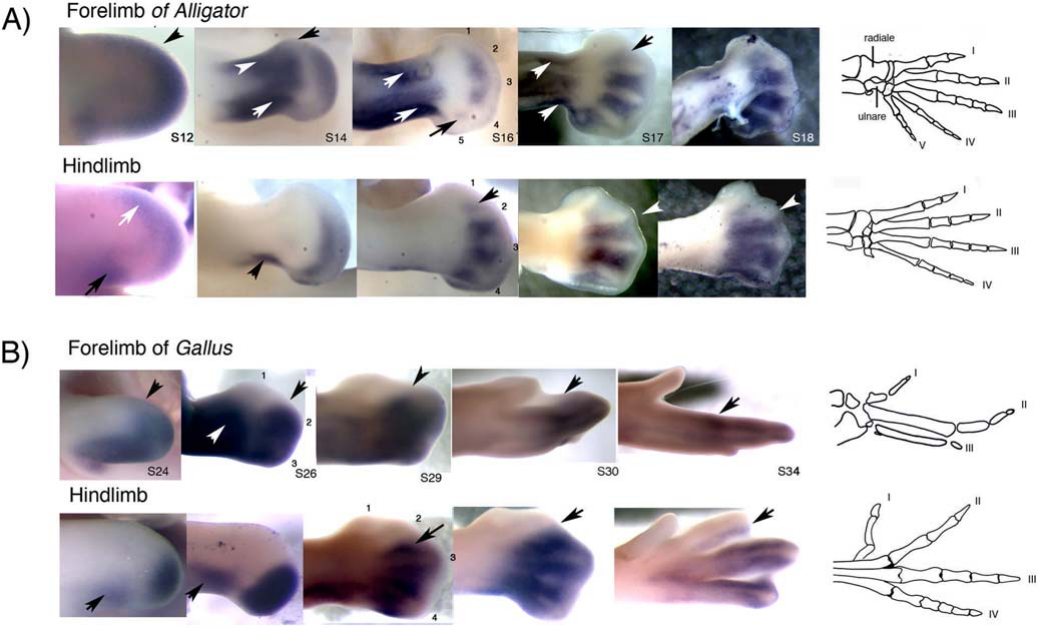
The expression of *HoxD-11* in alligator and chicken limbs. A) The expression of *HoxD-11* in the developing forelimb and hind limb of the crocodilian *Alligator mississippiensis* (staging is according to Ferguson [29]) B) The expression of *HoxD-11* in the chicken *Gallus gallus* (staging according to Hamburger-Hamilton [30]). Alligator forelimb: Early stages 12–14 show extension of *HoxD-11* along the limb border, including anterior regions (black arrows). At stage 16 and onwards, there is no detectable expression in the anterior-most digit 1 region. At stage 16 posterior expression is temporarily down-regulated in the region of digits 4 and 5 (black arrow) but is re-expressed by stage 17. At stage 17, only very low expression is detectable in the interdigit between digit 1 and 2 (black arrow). Strong anterior and posterior expression in the wrist and forearm region at stage 14 continues up to stage 17 (white arrows). Alligator hind limb: Stage 12 shows some expression along the anterior margin (white arrow) but this is undetectable in the anterior-most digit 1 region from stage 14 onwards. At stage 16, only very low expression is detectable in the interdigit between digits 1 and 2 (black arrow). Expression in the foreleg is restricted to posterior (stages 12 and 14, black arrow) and absent in the ankle region. Stage 17 and stage 18 show a sharp anterior limit of *HoxD-11* expression along the posterior margin of digit 2 (white arrows). Chicken wing: Stage 24 presents anterior expression (black arrow). Expression is undetectable in digit 1 in stage 26 and subsequent stages. No low expression is detectable between interdigits 1 and 2. In stages 26–34, expression of *HoxD-11* extends to the anterior border of digit 2 (black arrows). Chicken hind limb: Early stage 24 shows no anterior expression. No expression is ever detected in digit 1 precursor cells or the interdigit between digit 1 and digit 2. In stages 29–34 *HoxD-11* shows a sharp anterior limit along the posterior border of digit 2 (black arrows in stage 17–18 of alligator and stages 31–34 of chicken; stage 34 is shown in ventral view). Strong expression is found in the interdigit between digits 2 and 3 (stage 29, black arrow), as in forelimbs. *HoxD-11* expression in alligator limbs is consistent with the notion that, as in chicken, normal digit 1 determination occurs at late stages with absent or very low *HoxD-11* expression.

A genomic fragment was amplified by PCR with a primer pair targeting the conserved 5′ sequence of the *HoxD-11* coding sequence and a part of the homeobox (see Material and Methods). These primers target a sequence that corresponds to nucleotides 22 to 690 of the chicken *HoxD-11* coding sequence, but include the intron between exon 1 and 2. We obtained a PCR product of approximately 900 nucleotides and sequenced 819 nucleotides from the 5′ end of this sequence. This sequence contains the complete exon 1 of 606 nucleotides and the adjacent intron sequence with a putative 5′ splice site AG/GTAGGT (the G/G is the putative exon-intron boundary). The translated exon 1 sequence has 87% sequence conservation with the corresponding part of the chicken *HoxD-11* gene (Figure 3A). A phylogenetic analysis of this and published paralog group 11 amino acid sequences reveals strong support for the hypothesis that the alligator sequence is a *HoxD-11* ortholog. Our sequence forms a well supported clade with the chicken *HoxD-11* sequence and together with the human *HoxD-11* sequence is separated by a well supported node from *HoxA-11* and *HoxC-11* sequences (Figure 3 B, C). Furthermore, *in situ* hybridization revealed expression in all structures where *HoxD-11* is known to be expressed in other amniotes, as can be observed in Figure 3D. The specimen is dissected to show the sharp anterior limit of hindgut expression (Figure 3D, 1) expression in the genital tubercle (Figure 3D, 2), distal tail (Figure 3D, 3) and limbs (Figure 3D 4, Figure 4). We thus conclude that we have isolated the exon 1 and 5′ part of the intron of alligator *HoxD-11* gene (Genbank accession # EU597806).

### Expression of *HoxD-11* in embryonic limbs of alligator

The expression pattern of *HoxD-11* in the embryonic limbs of the alligator is presented in Figure 4A alongside with that of *HoxD-11* in the chicken (Figure 4B) for comparison. In the early alligator forelimb bud (stage 12, n = 1), the expression of *HoxD-11* extends along most of the margin of the limb bud, including anterior regions indicated by the black arrow. Early mouse forelimbs show similar anterior extension of *HoxD-11* transcripts (see stage 10.5 photographs in [15], [16]). Anterior expression in alligator forelimbs persists into stage 14 (n = 2), presumably including cell precursors of digit 1 (black arrow). However, as development proceeds and digital rays become apparent, anterior expression of *HoxD-11* in alligator forelimb is down-regulated and becomes undetectable in digit 1, as observed in stage 16 (n = 3), stage 17 (n = 3) and stage 18 (n = 1). In the forelimbs of the chicken, and in forelimbs and hind limbs of the mouse [11], strong expression extends up to the anterior border of digit two, as indicated by black arrows in stages 26 to 34 of the chicken wing (Figure 4B). In the foot of both chicken and alligator, late *HoxD-11* expression is slightly more shifted towards posterior: The anterior limit of expression is found along the posterior border of digit 2 (white arrows in hind limb stages 17–18 of the alligator, black arrows for stages 30 and 34 of the chicken hind limb).

A phenomenon particular to the alligator forelimb is a transient down-regulation of *HoxD-11* expression in the posterior region of digits 4 and 5 at stage 16, as indicated by the black arrow. Expression is recovered by stage 17. In contrast, the posterior expression of *HoxD-11* is uninterrupted in the hind limb of the alligator, as well as both forelimbs and hind limbs of chicken and mouse. Digits 4 and 5 of the adult alligator forelimb are special in that they are unusually slender, have no claws, and are proportionally reduced compared to reptilian outgroups such as lizards (specially digit 4; see Lepidosauria, Figure 1 node D). Assuming that *HoxD-11* expression affects growth rates [17], we can hypothesize that transient down-regulation of *HoxD-11* may relate to the partial reduction of these digits.

In all forelimbs examined. i.e. alligator, chicken, and mouse, there is early expression of *HoxD-11* in the mesopodial (“wrist”) and zeugopodial (“forearm”) regions [11], [13] (Figure 4A). In alligator, strong anterior and posterior expression in the mesopodial-zeugodial region of the forelimb persists into late stages (white arrows in Stages 14, 16 and 17). In the chicken and alligator hind limb, mesopodial expression is absent, and zeugopodial expression is found in a posteriorly restricted domain (black arrow in alligator stage 14 and chicken stage 26 hind limbs). In the mouse hind limb, mesopodial expression is present, but weaker than in forelimbs [13]. This expression in the wrist of alligator may relate to the unusual lengthening of the radiale and ulnare wrist-bones, a derived trait of crocodilians (Figure 4A, adult skeleton). This difference is reminiscent of the stronger *HoxA-11* expression in the frog hind limb compared to the forelimb, where the frog hind limb also has a pair of elongated mesopodial bones [18].

## Discussion

### Expression of *HoxD-11* in alligator digits is consistent with its function in other amniotes

When cartilaginous digital rays are formed they are at first undetermined. Digital identity becomes fixed at fairly late stages under the influence of the surrounding tissues (HH26–30 in the chicken foot) [19], [20], [21]. Here we will be concerned with late-phase gene expression in the mesenchyme surrounding digital rays during digit determination. At these late stages, the digital ray corresponds mostly to the future metacarpal (or metatarsal); only the distal most tip gives rise to phalanges, at the PFR (Phalanx Forming Region) [21]. The mesenchyme immediately posterior to the PFR is crucial to phalangeal number and morphology. Small heterotopic grafts of interdigital mesenchyme pinned into this region are sufficient to cause homeotic transformations of digits in the chicken foot [21]. In mouse and chicken limbs, late phase expression of *HoxD-10*, *HoxD-11* and *HoxD-12* is absent only in digital ray 1 and the interdigital mesenchyme between digit 1 and digit 2, but these are strongly expressed elsewhere. Forced expression of *HoxD-12* in the entire mouse limb frequently leads to the transformation of digit 1 into a triphalangeal digit 2 [22]. In the chicken, forced expression of *HoxD-11* in the entire hind limb often leads to the transformation of digit 1 into digit 2 [23]

Late phase-expression of *HoxD-11* in the alligator forelimb is consistent with a similar role for *HoxD-11* as in other amniotes. The transient expression in precursor cells of digit 1 in alligator forelimbs appears too early to participate in digit determination. Transient anterior expression is also apparent in early chicken and mouse forelimbs. In the chicken foot, digit 1 is the last digit to be determined, at stage 30 [21] (See Figure 4B). *HoxD-11* is absent in digit 1 of alligator forelimbs at a comparable late stage (Figure 4A, stage 17). Our *in situs* also reveal some weak expression in the interdigital mesenchyme between digit 1 and digit 2 of alligator limbs (indicated by the arrow in stage 17 alligator forelimb and stage 16 of the hind limb, Figure 4). Very low expression levels of *HoxD-11* in interdigit 1 may not be mechanistically relevant, specially in the case of HoxD genes, who show functional overlap and quantitative, additive effects [9], [10]. Importantly, there is no expression in the mesenchyme immediately posterior to the distal phalanx-forming region (PFR) of digit 1 in alligator, unlike strong expression for digit 2. Expression in the alligator suggests that, as in mouse and chicken limbs, digit 1 determination normally occurs in absence of *HoxD-11* expression.

### The asymmetric late *HoxD-11* expression related to “thumbness” is conserved in alligator

Late phase expression of *HoxD-11* is asymmetric in the pectoral fins of the basal bony fish *Polyodon spathula*, with no expression present in the anterior most region of developing fin radials [24]. The autopod (digit ray region) of amniotes shows similar late asymmetric expression related to “thumbness” [25], [26], with no anterior expression in digit 1. Hence an anterior autopodial domain of no *HoxD-11* expression is an ancient marker of positional identity along the anterior-posterior axis of paired appendages. As expected, in alligator lack of expression of *HoxD-11* was found only at the biphalangeal digit 1 of both the forelimb and hind limb. Adding crocodilians to the comparison of mouse and chicken allows the inference of ancestral and derived expression patterns. The ancestral condition for amniotes is observed in the mouse and the alligator: The anterior limit of late-phase *HoxD-11* expression does not extend into the biphalangeal digit 1, and is separated by two digital positions from the primary axis. In the wing of birds, the development of a biphalangeal digit in absence of *HoxD-11* expression is conserved, but the embryological position of *HoxD-11* expression is derived, a full digital position closer to the primary axis (Figure 1C). The shifted morphology and *HoxD-11* expression suggests both were effected by a mechanism upstream of *HoxD-11* and other HoxD genes. Our analysis is consistent with that of other authors that have argued the primary axis in the bird wing exceptionally develops into digit 3, rather than digit 4 [6], [28], [29]. To further understand the role of *HoxD-11* expression in the evolution of digits, we encourage the study of more taxa across a broad taxonomic sample, including other species where morphology also seems to have shifted embryological position, for instance the three-toed Italian skink, *Chalcides chalcides* [2], [29]

## Materials and Methods

### Cloning and sequencing of an exon 1 fragment from the alligator *HoxD-11* gene

DNA extractions were performed on alligator embryo samples preserved in 95% ethanol using the DNAEasy Tissue kit (Qiagen inc) according to the manufacturer's protocol. Degenerate primers for *HoxD-11* were designed targeting the conserved 5′ region of exon one and part of the homeo box.

1FD11: ATGAMCGASTTTGACGAKTGC

1RD11: CKTTTCTCTTTGTTTATGTABAC

PCR was performed with a range of annealing temperatures between 46 and 48°C. The resulting PCR product was about 900 bp long and cloned. Several clones were sequenced in order to evaluate PCR errors. To confirm its identity as *HoxD-11* the sequence was analyzed using Neighbor Joining and Likelihood analysis as implemented in Phylip (http://evolution.genetics.washington.edu/phylip.html). To prepare an *in situ* probe, we cloned a fragment of exon 1 into a bacterial plasmid vector that was used to transcribe a labeled anti-sense mRNA probe.

### Egg collection

The *Alligator mississippiensis* eggs were collected from the Rockefeller Wildlife Refuge in southwestern Louisiana in June of 2006. Under the direction of refuge biologist Ruth Elsey, J. V. collected recently deposited eggs. To reveal the eggs, the top layer of vegetation was removed and set aside. At the nest, all of the eggs were removed one by one and carefully marked with a black pencil to indicate the side of the egg that was oriented toward the top of the nest. This is necessary because if the eggs are turned over, the embryos contained inside run a high risk of drowning in the albumen or becoming detached from the top membrane of the egg, which shortly leads to death of the embryo. The eggs were then transported to Yale University, in wire mesh cages containing vegetation collected directly from the nest.

### Incubation

The eggs were incubated in Plexiglas aquaria (30″×12″×12″) covered with machined Plexiglas lids with holes cut for ventilation. The incubators were filled with tap water to a depth of five inches. RenaCal™ Basic 100 Watt aquarium heaters were inserted under the water and set to a temperature of ∼31.5°C to regulate the internal temperature of the incubators as well as create a high humidity atmosphere within the incubator. To create a platform for the eggs, two 6″ high×12″ long drying racks that together covered most of the length of the incubators while leaving gaps for water to evaporate through were placed on the bottom of the aquaria. This setup left a space of 1″ between the top of the water and the top of the drying racks. A layer of nesting material that was brought back from the alligator nests was placed on top of the drying racks. The eggs were transferred from the cages and rested on top of this layer of vegetation. A second layer of nesting materials was then used to cover the eggs. The natural nesting material is ideal for insulation of the eggs and allowing them to stay moist, but not wet. The relative humidity level was regulated to approach 100%. The incubators were kept in a temperature and humidity controlled animal care room, in which the temperature was set at 31°C and the relative humidity at 60%. Within the incubators, the temperature was held constant at 31.5°C and the relative humidity was held at near 100%. Because of approximately 1–2 weeks of uncertainty regarding the date when eggs were laid, staging was done according to the embryological series and stages described in [30]

Chicken fertilized eggs were obtained from Charles Rivers Laboratories and incubated in polysterene egg incubators (Hova-bator) at 37°C with a water tray for humidity. Embryos were collected at 12-hour intervals, staged (according to [31]) and fixed in fixed overnight in 4% paraformaldehyde, rinsed in PBS, dehydrated in a sequence of methanol concentrations and preserved in methanol 100% at −20°C.

### In situ hybridization

Alligator embryos were collected at 1-day intervals and fixed, dehydrated and stored as with chicken embryos above. Antisense probes for chicken and alligator *HoxD-11* labeled with digoxigenin were prepared to visualize the transcripts of these genes in the developing limbs. In situ hybridization was carried out following standard procedures described in [32]. Chicken *HoxD-11* plasmid was obtained from C. Tabin's laboratory. No special modifications of the standard protocol were required to successfully perform in situ hybridization on alligator embryos.
